# Supplementary material for: Sense of doubt: inaccurate and alternate locations of virtual magnetic displacements may give a distorted view of animal magnetoreception ability
Source: Commun Biol. 2023 Feb 20;6:187. doi: 10.1038/s42003-023-04530-w (PMC9941108; doi:10.1038/s42003-023-04530-w)
Supplement: Supplementary file 2 — Supplementary Information [file 42003_2023_4530_MOESM2_ESM.pdf]

Supplementary material for Sense of Doubt Manuscript - Review of all bi-coordinate virtual magnetic displacement studies.

Twenty-three studies were found that used bi-coordinate virtual magnetic displacements with multiple possible locations, and one study was found using a tri-coordinate virtual magnetic displacement that existed at multiple possible locations. Each of these are shown on the pages below using our MATLAB tool 'ViMDAL' (except those already discussed in the main text of the article). International Geomagnetic Reference Field (IGRF) values were used for all visualisations, and dates were made to match those used in each study. In some cases, authors used manual measurements rather than IGRF values which can account for some discrepancies between intended and visualised location.

Birds

1. (Boström et al., 2010) – in main article.
2. (Boström et al., 2012) – Northern magnetic displacements trigger endogenous fuelling responses in a naïve bird migrant

Two magnetic parameters used (inclination and totally intensity). Two of the displacements are exactly the same as the previous Bostrom paper, but the experiment was conducted at a later date. Possible locations with the same value of inclination and intensity as the natural site (blue area) also appear out into the Atlantic Ocean. Possible locations for the first virtual displacement (orange area) also exist in South East Europe.

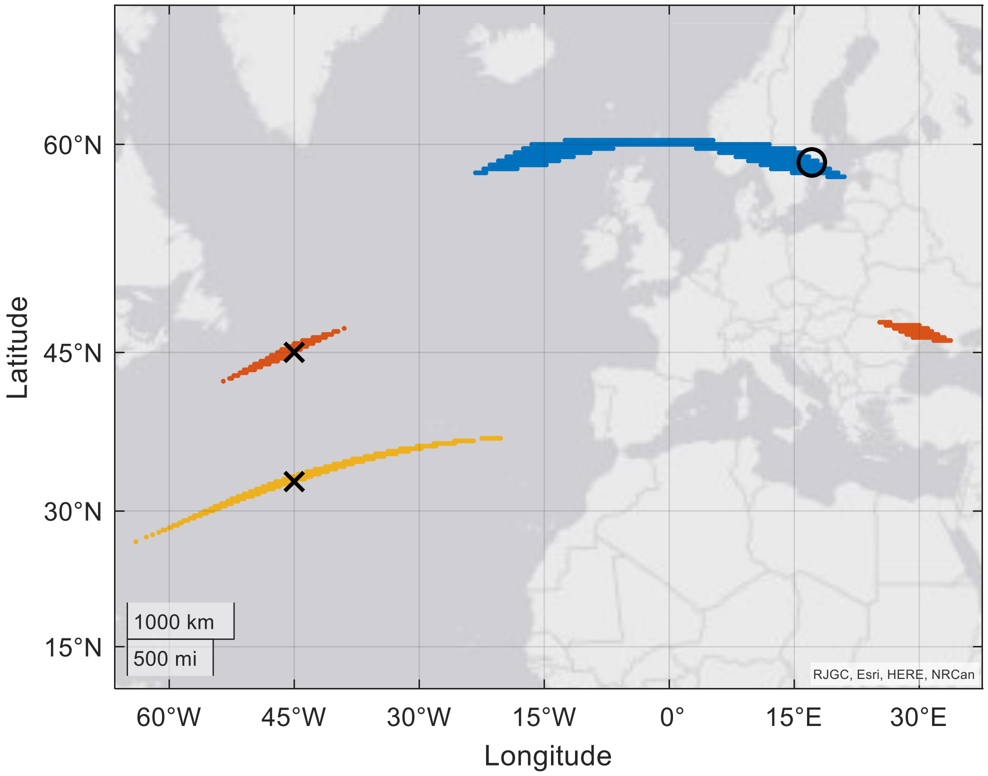

Supplementary Figure 1. Possible locations for the virtual magnetic displacement in Bostrom et al., 2012. Virtual magnetic displacement locations as suggested by the authors are indicated with 'x'. Control test location under the ambient magnetic field is indicated with 'o'. Possible locations with the same magnetic parameters as the virtual magnetic displacements and/or ambient conditions are highlighted in individual colours for each. The sensitivity used is ± 200 nT for total intensity, and ± 0.5° for inclination.

3. (Henshaw et al., 2008) - Food intake and fuel deposition in a migratory bird is affected by multiple as well as single-step changes in the magnetic field

Two magnetic parameters used (inclination and total intensity). Food intake assessed at home site under natural conditions, and at a virtual magnetic displacement intended to be located in Egypt. Possible locations with the same values of inclination and intensity as the natural site (blue area) also appear out into the Atlantic Ocean. Possible locations for the virtually displaced treatment are well confined to the intended location in Egypt (orange area).

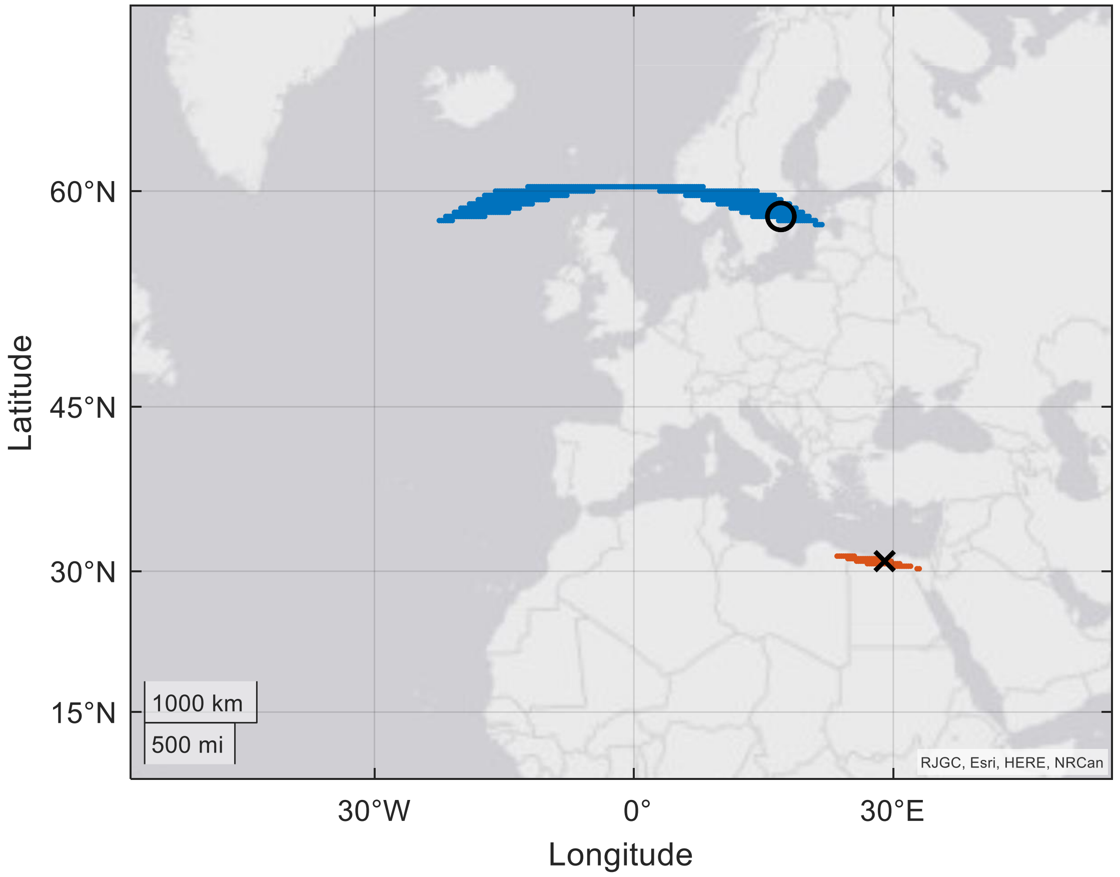

Supplementary Figure 2. Possible locations for the virtual magnetic displacement in Henshaw et al., 2008. Virtual magnetic displacement location as suggested by the authors is indicated with 'x'. Control test location under the ambient magnetic field is indicated with 'o'. Possible locations with the same magnetic parameters as the virtual magnetic displacements and/or ambient conditions are highlighted in individual colours for each. The sensitivity used is ± 200 nT for total intensity, and ± 0.5° for inclination.

4. (Henshaw et al., 2009) - Information from the geomagnetic field triggers a reduced adrenocortical response in a migratory bird

Similar to Henshaw et al., 2008, but cortisol also measured. Two magnetic parameters used (inclination and intensity). One test under natural conditions, another in a virtual displacement intended to be located in Egypt. Possible locations with the same values of intensity and inclination also as the natural condition also exist in the Atlantic Ocean (blue area). Possible values with the same intensity and inclination as the virtual displacement do not appear near the intended location, and instead occur a significant distance Westwards in the mid-Atlantic (orange area).

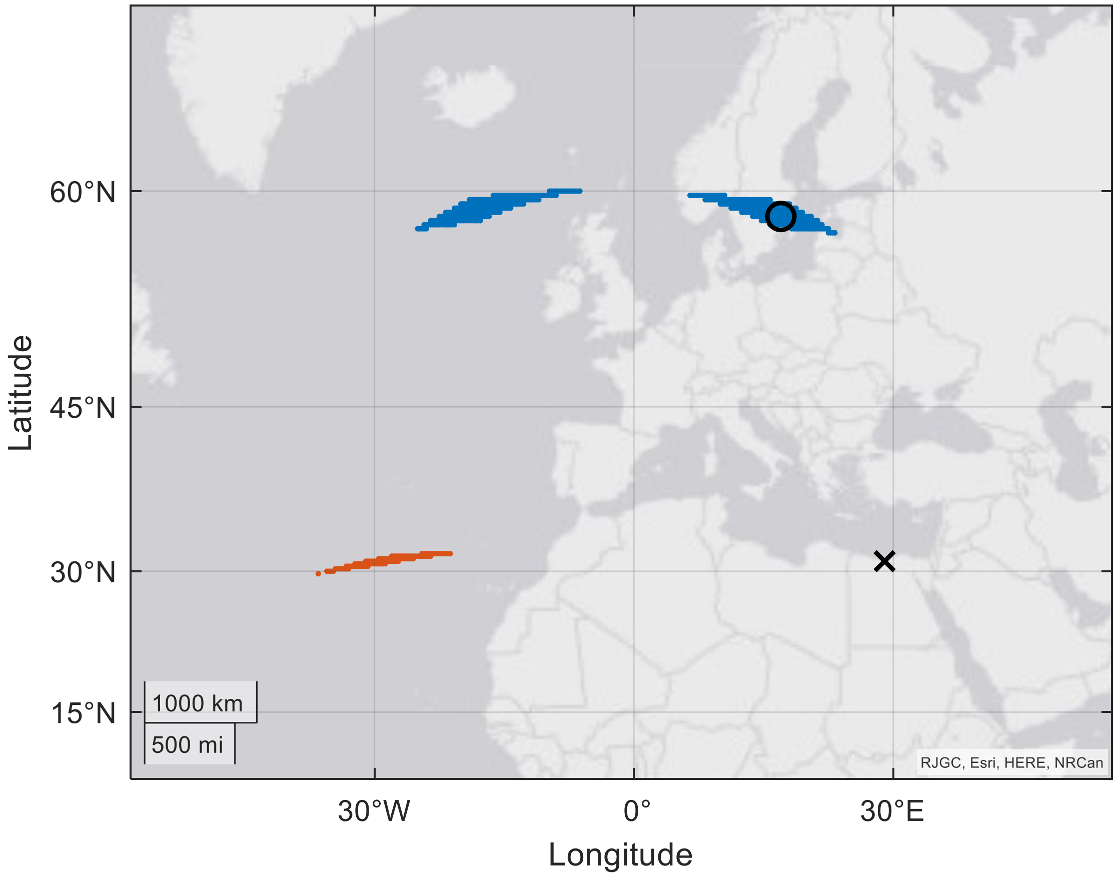

Supplementary Figure 3. Possible locations for the virtual magnetic displacement in Henshaw et al., 2009. The virtual magnetic displacement location as suggested by the authors is indicated with 'x'. Control test location under the ambient magnetic field is indicated with 'o'. Possible locations with the same magnetic parameters as the virtual magnetic displacements and/or ambient conditions are highlighted in individual colours for each. The sensitivity used is ± 200 nT for total intensity, and ± 0.5° for inclination.

5. (Henshaw et al., 2010) - Geomagnetic field affects spring migratory direction in a long-distance migrant

Two magnetic parameters used (inclination and intensity). Orientations assessed under two virtual displacements, one intended in Northern Norway, and the other in Czechia. Possible locations with same values of intensity and inclination appear elsewhere of the intended locations for both virtual magnetic displacements (orange and purple areas).

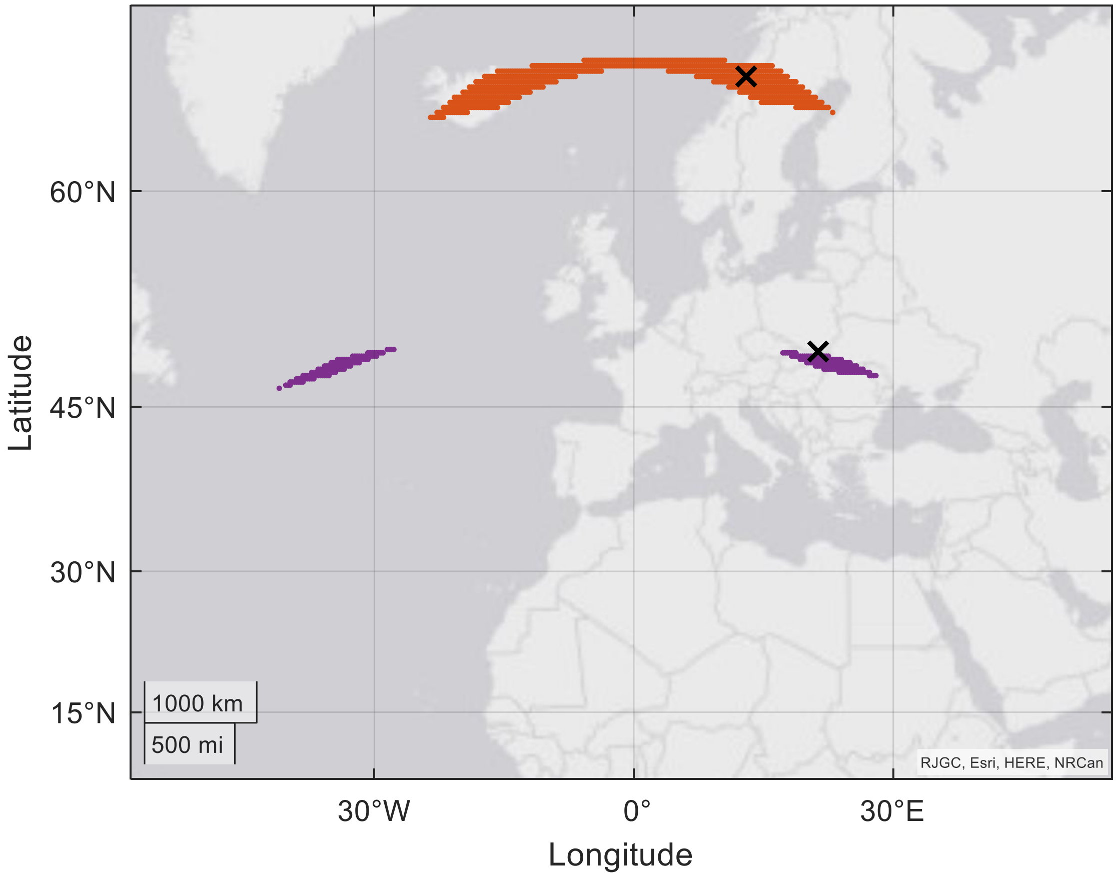

Supplementary Figure 4. Possible locations for the virtual magnetic displacements in Henshaw et al., 2010. Virtual magnetic displacement locations as suggested by the authors are indicated with 'x's. Possible locations with the same magnetic parameters as the virtual magnetic displacements and/or ambient conditions are highlighted in individual colours for each. The sensitivity used is ± 200 nT for total intensity, and ± 0.5° for inclination.

6. (Ilieva et al., 2016) – Does migratory distance affect fuelling in a medium-distance passerine migrant?: results from direct and step-wise simulated magnetic displacements

Two magnetic parameters used (intensity and inclination). Migratory activity assessed under natural field and ten virtual magnetic displacements, located North and South of test site. Possible locations with the same values of intensity and inclination span wide areas Westwards for all virtual magnetic displacements.

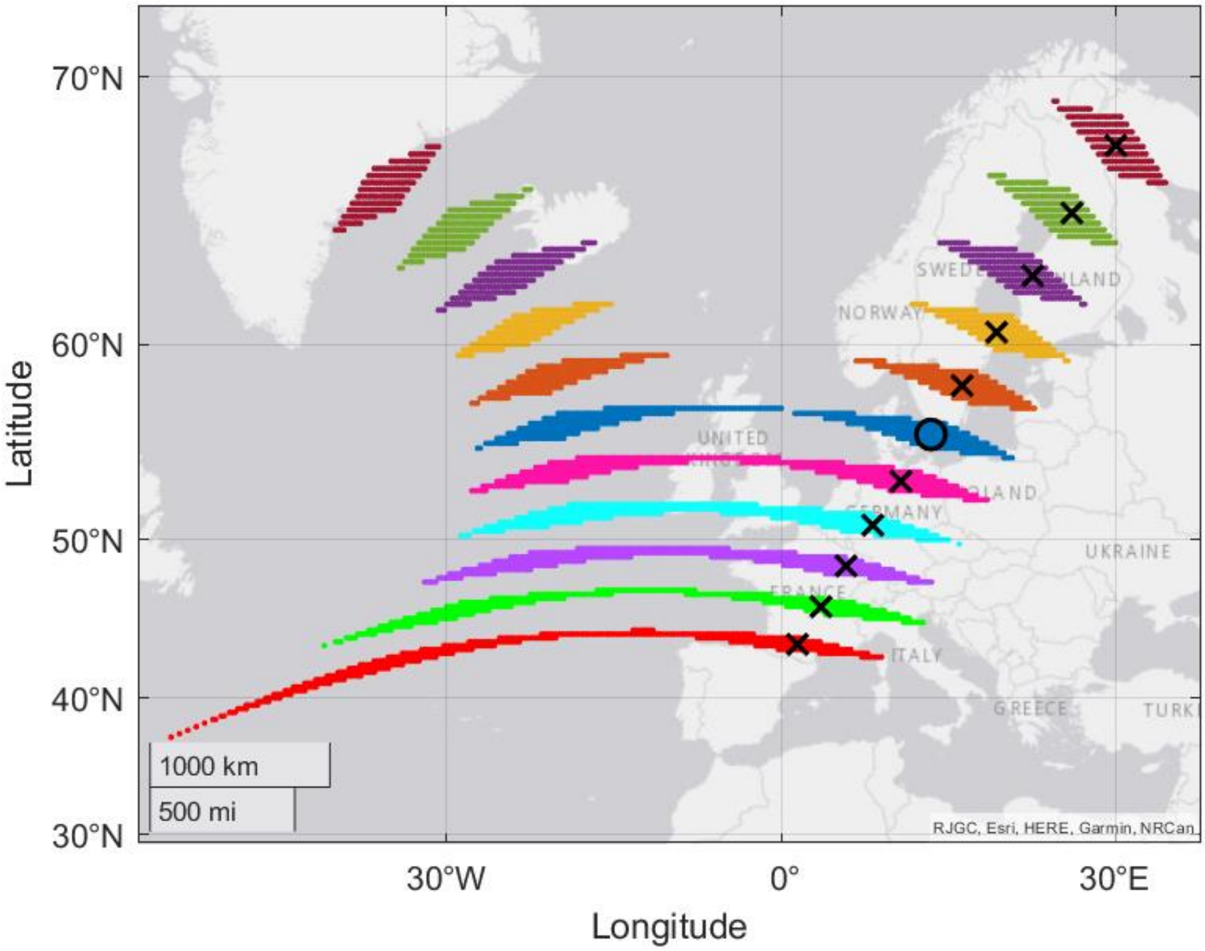

Supplementary Figure 5. Possible locations for the virtual magnetic displacements in Ilieva et al., 2016. Virtual magnetic displacement locations as suggested by the authors are indicated with ‘x’s. Control test location under the ambient magnetic field is indicated with ‘o’. Possible locations with the same magnetic parameters as the virtual magnetic displacements and/or ambient conditions are highlighted in individual colours for each. The sensitivity used is ± 200 nT for total intensity, and ± 0.5° for inclination.

7. (Ilieva et al., 2018) - Effect of geomagnetic field on migratory activity in a diurnal passerine migrant, the dunnock, *Prunella modularis*

Two magnetic parameters used (intensity and inclination), much the same as previous Ilieva paper. Migratory activity assessed under natural field and ten virtual magnetic displacements, located North and South of test site. Possible locations with the same values of intensity and inclination span wide areas Westwards for all virtual magnetic displacements.

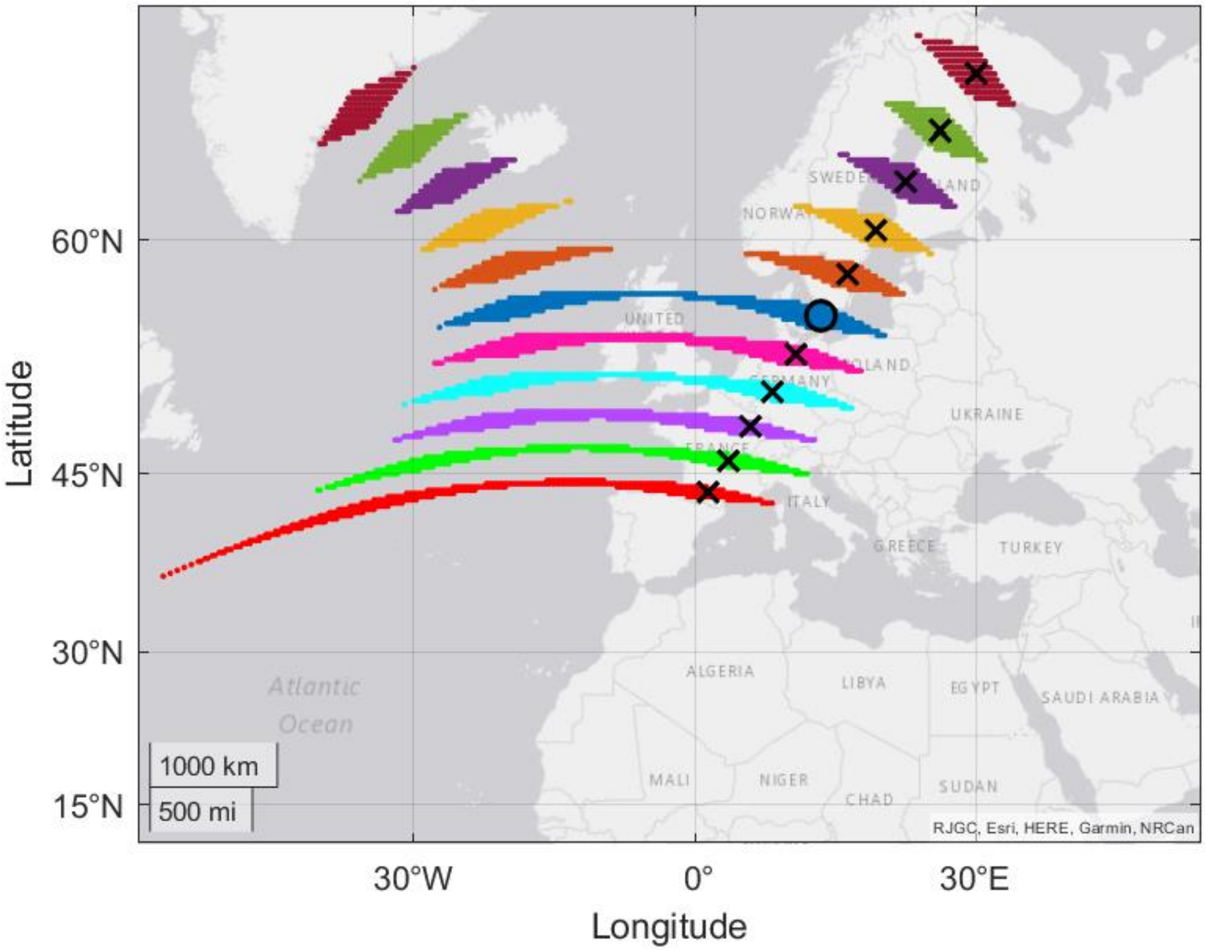

Supplementary Figure 6. Possible locations for the virtual magnetic displacements in Ilieva et al., 2018. Virtual magnetic displacement locations as suggested by the authors are indicated with ‘x’s. Control test location under the ambient magnetic field is indicated with ‘o’. Possible locations with the same magnetic parameters as the virtual magnetic displacements and/or ambient conditions are highlighted in individual colours for each. The sensitivity used is ± 200 nT for total intensity, and ± 0.5° for inclination.

8. (Kullberg et al., 2003) Magnetic cues and time of season affect fuel deposition in migratory thrush nightingales

Two magnetic parameters used (intensity and inclination). Fuel deposition assessed for control birds and for experimentally virtually displaced birds (three virtual displacements, exact locations for intermediate displacements not given). Possible locations with the same values of intensity and inclination span areas Westwards, except for the virtual magnetic displacement located in Egypt.

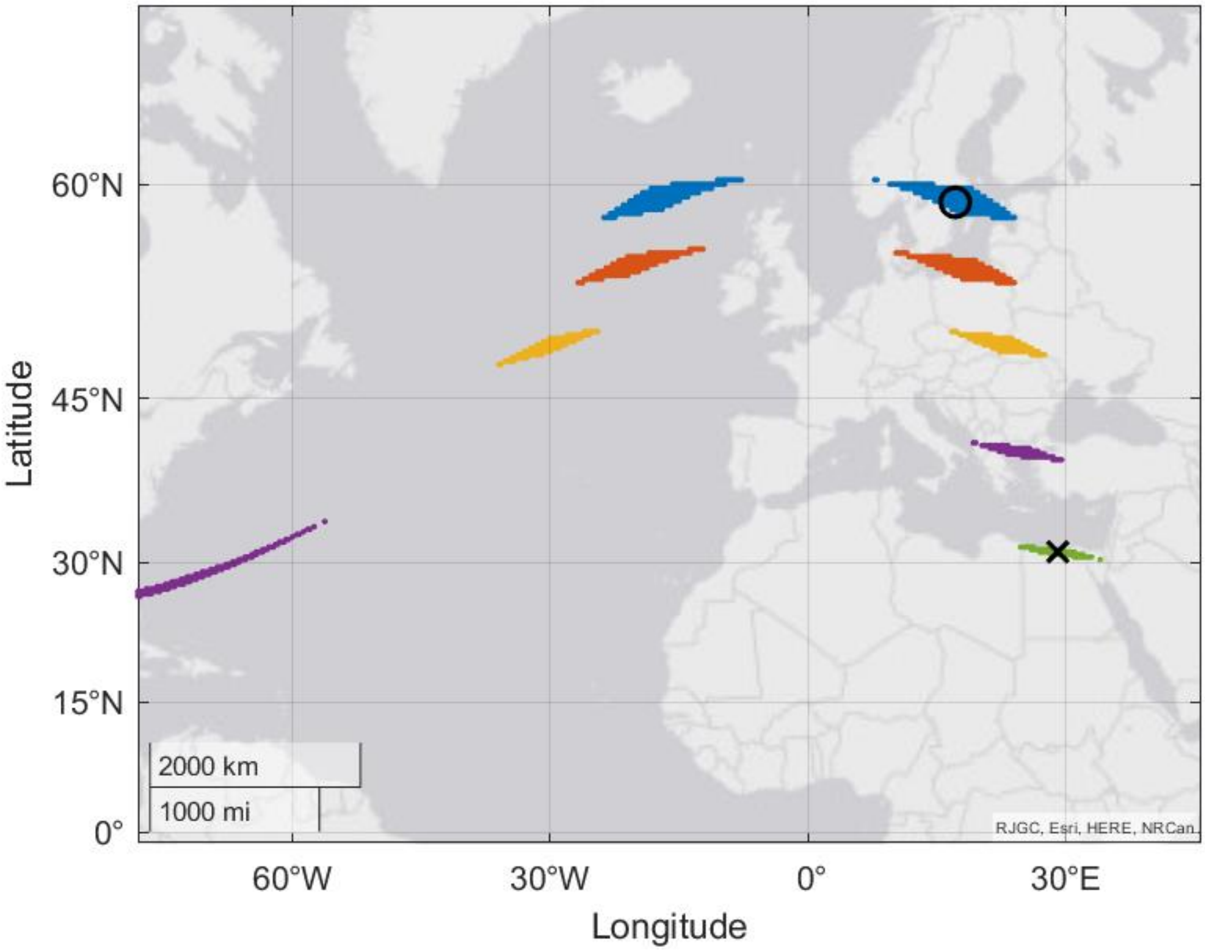

Supplementary Figure 7. Possible locations for the virtual magnetic displacements in Kullberg et al., 2003. Virtual magnetic displacement locations for the final displacement is indicated with 'x', locations for the other virtual displacements are not detailed in the paper. Control test location under the ambient magnetic field is indicated with 'o'. Possible locations with the same magnetic parameters as the virtual magnetic displacements and/or ambient conditions are highlighted in individual colours for each. The sensitivity used is ± 200 nT for total intensity, and ± 0.5° for inclination.

9. (Kullberg et al., 2007) Fuelling decisions in migratory birds: geomagnetic cues override the seasonal effect

Two magnetic parameters used (intensity and inclination). Fuel deposition assessed over a series of six virtual magnetic displacements. Possible locations with same values of inclination and intensity span Westwards for all virtual magnetic displacements and for control site.

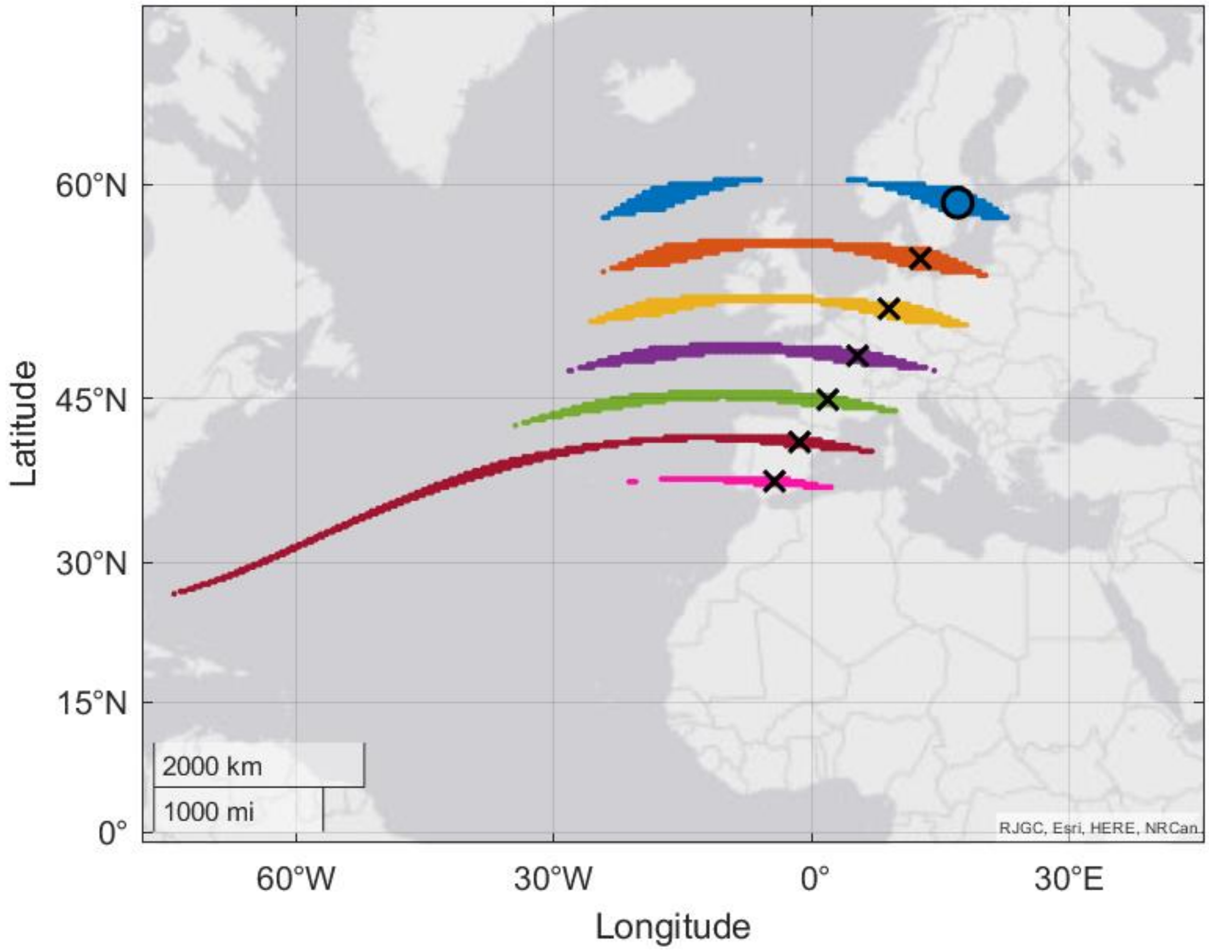

Supplementary Figure 8. Possible locations for the virtual magnetic displacements in Kullberg et al., 2007. Virtual magnetic displacement locations as suggested by the authors are indicated with 'x's. Control test location under the ambient magnetic field is indicated with 'o'. Possible locations with the same magnetic parameters as the virtual magnetic displacements and/or ambient conditions are highlighted in individual colours for each. The sensitivity used is ± 200 nT for total intensity, and ± 0.5° for inclination.

10. (Bulte et al., 2017) – Geomagnetic information modulates nocturnal migratory restlessness but not fueling in a long distance migratory songbird

Three magnetic parameters used (intensity, inclination, and declination). Migratory restlessness was assessed in control birds vs birds that were given sequential virtual magnetic displacements from Norway to West Africa. Possible locations for one of these displacements (purple) exist in the Gulf of Mexico, and for three displacements (purple, green, maroon), possible locations also exist in the Western Pacific Ocean. No possible locations exist for one of the virtual magnetic displacements.

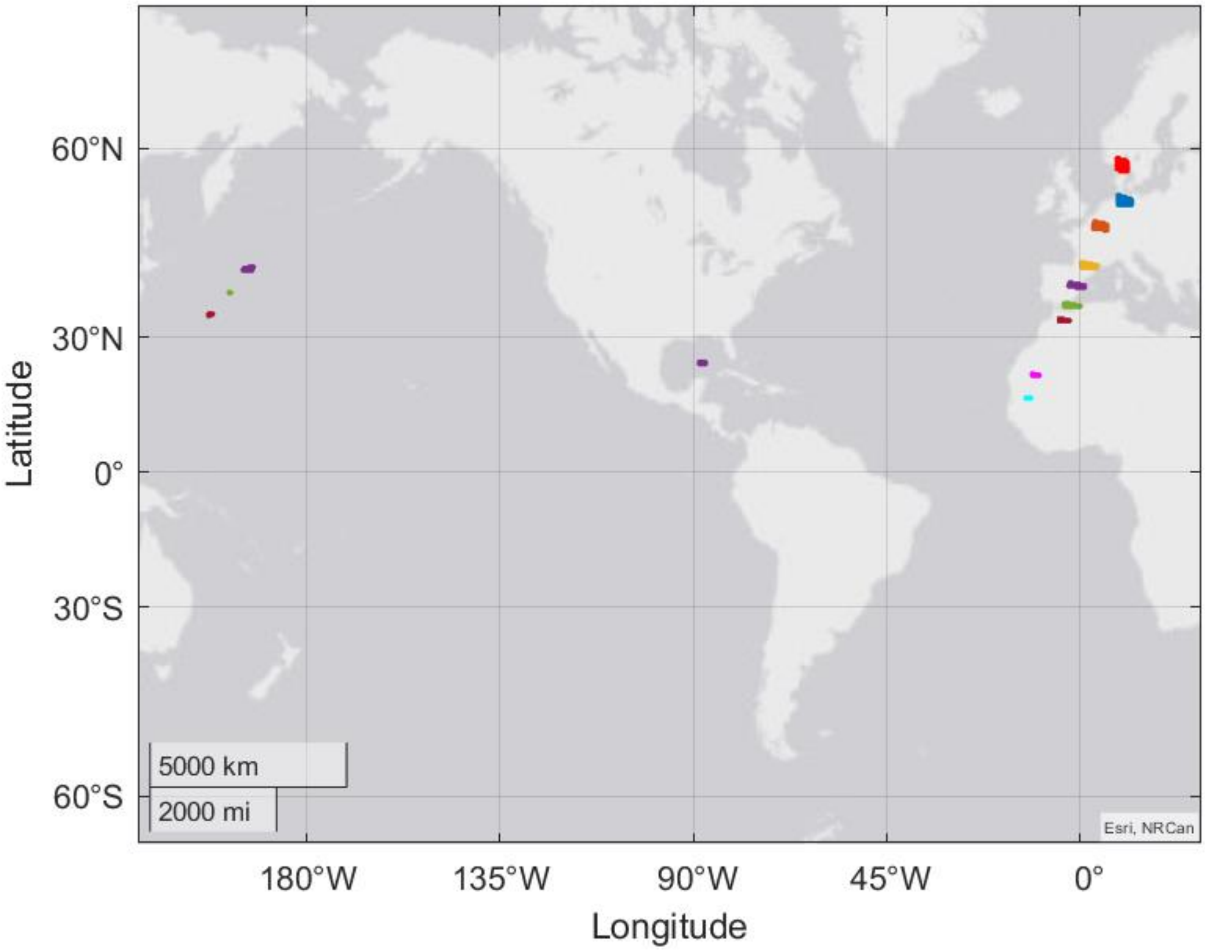

Supplementary Figure 9. Possible locations for the virtual magnetic displacements in Bulte et al., 2017. Latitude and longitude locations for the virtual magnetic displacements were not detailed in the paper. Possible locations with the same magnetic parameters as the virtual magnetic displacements and/or ambient conditions are highlighted in individual colours for each. The sensitivity used is ± 200 nT for total intensity, and ± 0.5° for inclination and declination.

Fish

11. (Keller et al., 2021) Map-like use of Earth's magnetic field in sharks.

Two magnetic parameters used (intensity and inclination). Orientation assessed at a control location and two virtual magnetic displacements. Possible locations with the same values of inclination and intensity span a wide area East and West for the control location and the Southern virtual displacement. No possible locations exist for the magnetic parameters used that should correspond to the Northern displacement.

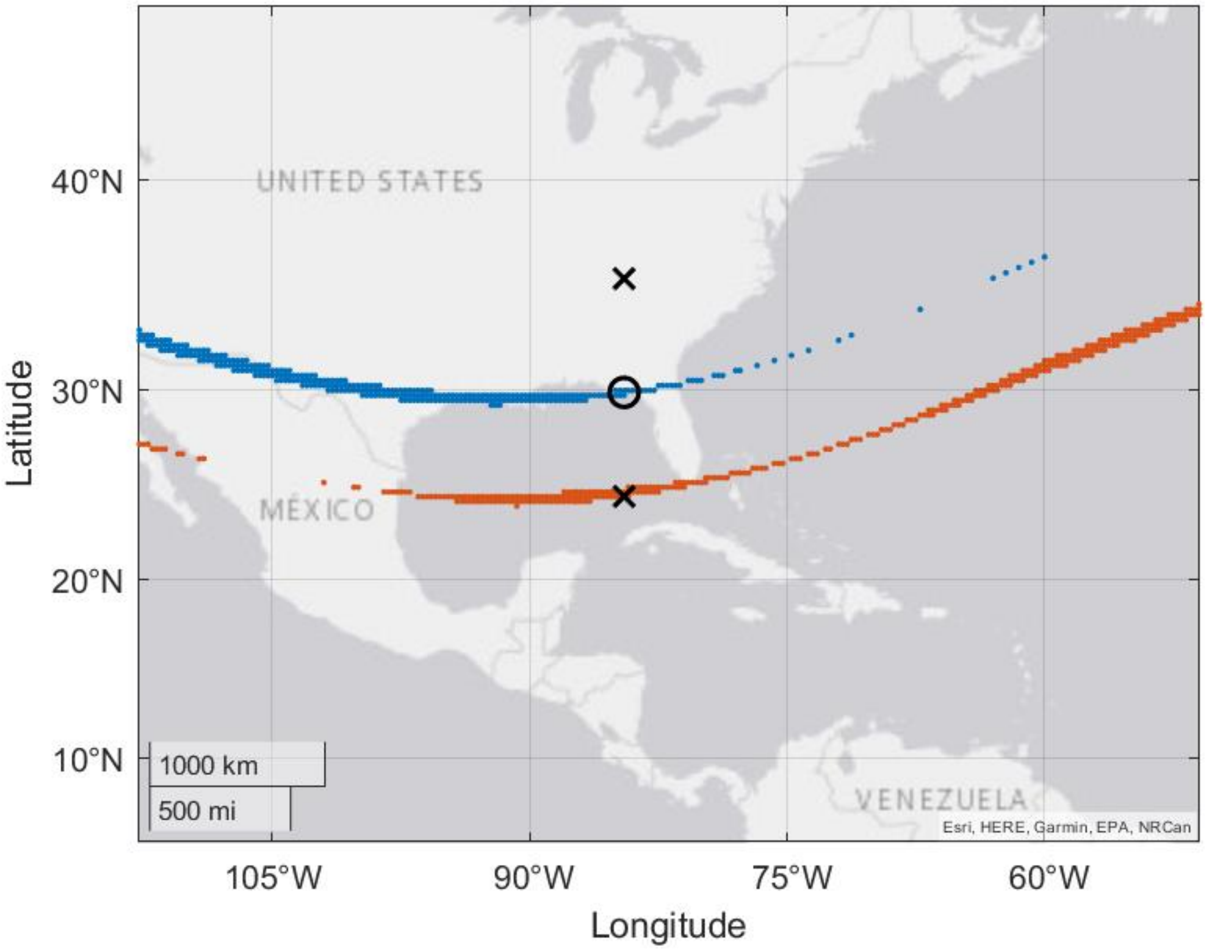

Supplementary Figure 10. Possible locations for the virtual magnetic displacements in Keller et al., 2021. Virtual magnetic displacement locations as suggested by the authors are indicated with 'x's. Control test location under the ambient magnetic field is indicated with 'o'. Possible locations with the same magnetic parameters as the virtual magnetic displacements and/or ambient conditions are highlighted in individual colours for each. The sensitivity used is ± 200 nT for total intensity, and ± 0.5° for inclination.

12. (Naisbett-Jones et al., 2017) A Magnetic Map Leads Juvenile European Eels to the Gulf Stream

Two magnetic parameters used (inclination and intensity). Orientation assessed at ambient conditions at control site and at three virtual magnetic displacements. Possible locations for the given magnetic parameters do not match up well with the suggested locations, except for the virtual magnetic displacement on the North American coast. The parameters provided for the virtual magnetic displacement in the Northern Mid-Atlantic are the same as those provided for the control site, though neither match up with the possible locations.

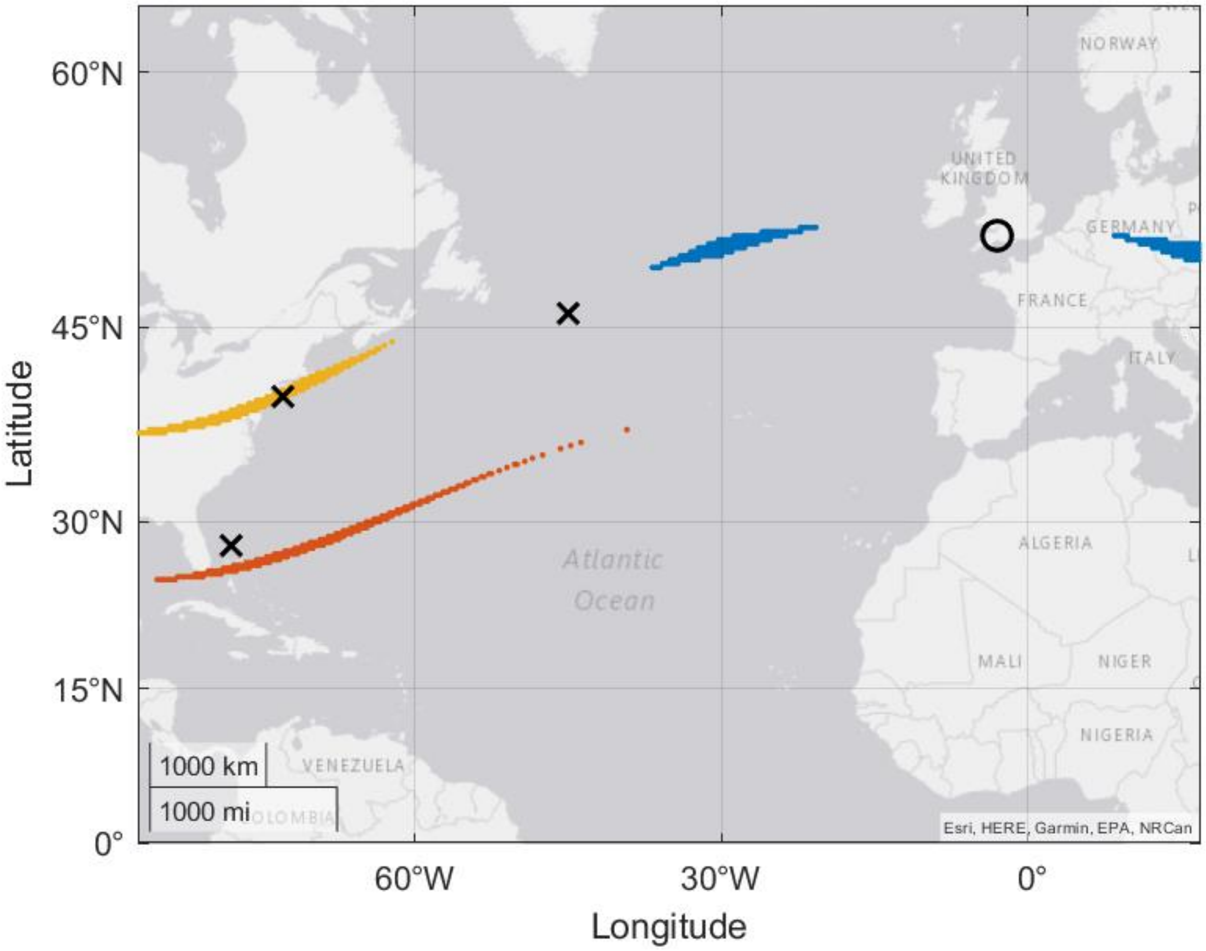

Supplementary Figure 11. Possible locations for the virtual magnetic displacements in Naisbett-Jones et al., 2017. Virtual magnetic displacement locations as suggested by the authors are indicated with 'x's. Control test location under the ambient magnetic field is indicated with 'o'. Possible locations with the same magnetic parameters as the virtual magnetic displacements and/or ambient conditions are highlighted in individual colours for each. The sensitivity used is ± 200 nT for total intensity, and ± 0.5° for inclination.

13. (Putman et al., 2020) A sense of place: pink salmon use a magnetic map for orientation

Two magnetic parameters used (inclination and intensity). Orientations assessed at ambient conditions and at two virtual magnetic displacements. Possible locations with the same magnetic parameters as the virtual displacements span wide areas across the Pacific Ocean, both sides of North America, and into the Atlantic Ocean.

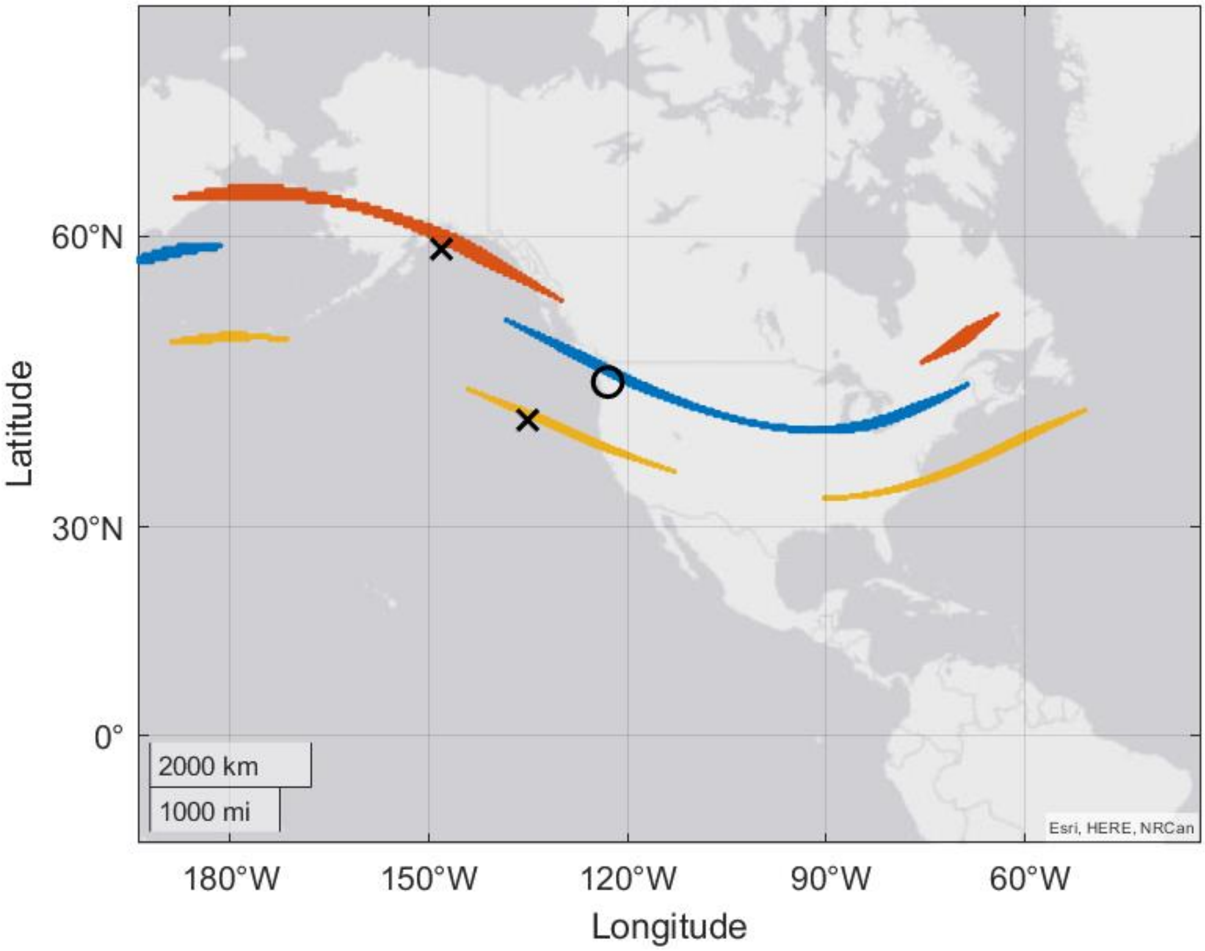

Supplementary Figure 12. Possible locations for the virtual magnetic displacements in Putman et al., 2020. Virtual magnetic displacement locations as suggested by the authors are indicated with ‘x’s. Control test location under the ambient magnetic field is indicated with ‘o’. Possible locations with the same magnetic parameters as the virtual magnetic displacements and/or ambient conditions are highlighted in individual colours for each. The sensitivity used is ± 200 nT for total intensity, and ± 0.5° for inclination.

14. (Putman et al., 2014a) Rearing in a distorted magnetic field disrupts the 'map sense' of juvenile steelhead trout.

Two magnetic parameters used (inclination and intensity). Orientations assessed at two virtual magnetic displacements. Possible locations for both span wide areas of the Pacific Ocean, and also on land into Russia and North East Canada for the Northern virtual magnetic displacement.

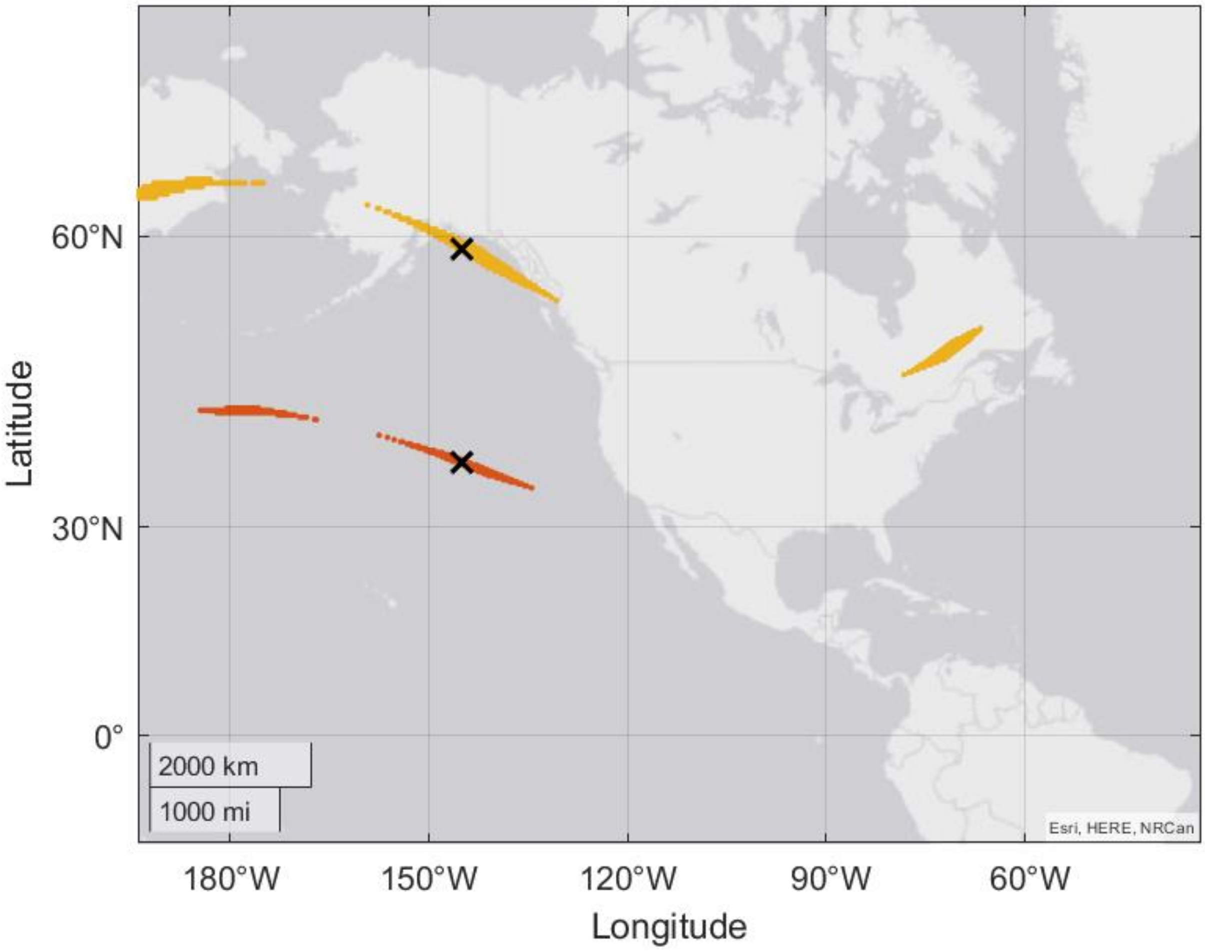

Supplementary Figure 13. Possible locations for the virtual magnetic displacements in Putman et al., 2014a. Virtual magnetic displacement locations as suggested by the authors are indicated with 'x's. Possible locations with the same magnetic parameters as the virtual magnetic displacements and/or ambient conditions are highlighted in individual colours for each. The sensitivity used is ± 200 nT for total intensity, and ± 0.5° for inclination.

15. (Putman et al., 2014b) An Inherited Magnetic Map Guides Ocean Navigation in Juvenile Pacific Salmon

Two magnetic parameters used (inclination and intensity). Orientations assessed at two virtual magnetic displacements and at the ambient field. Possible locations with the same magnetic parameters as the ambient field do not exist near to the suggested location. Many possible locations exist for the virtual magnetic displacements, spreading across the Pacific Ocean.

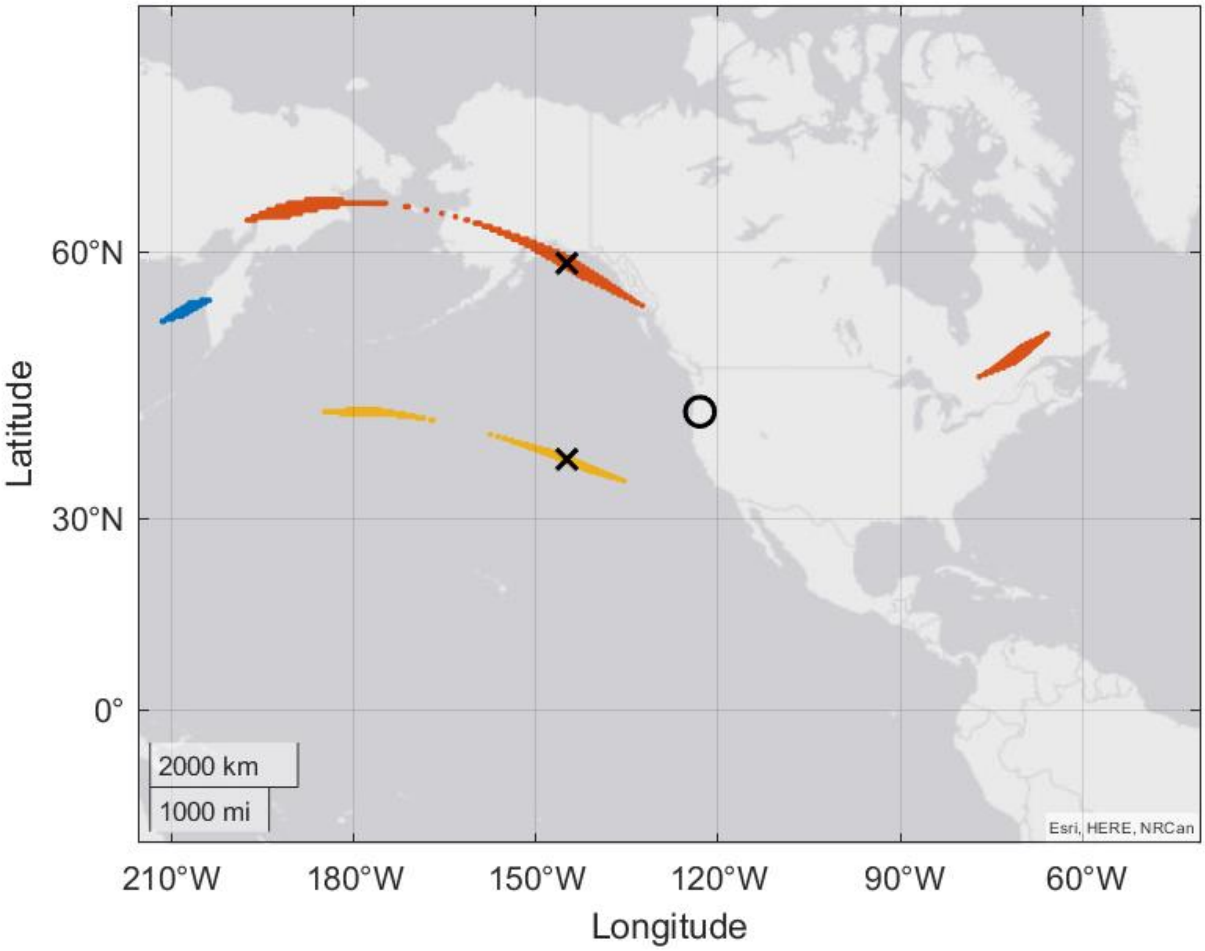

Supplementary Figure 14. Possible locations for the virtual magnetic displacements in Putman et al., 2014b. Virtual magnetic displacement locations as suggested by the authors are indicated with ‘x’s. Control test location under the ambient magnetic field is indicated with ‘o’. Possible locations with the same magnetic parameters as the virtual magnetic displacements and/or ambient conditions are highlighted in individual colours for each. The sensitivity used is ± 200 nT for total intensity, and ± 0.5° for inclination.

16. (Scanlan et al., 2018) – in main article.

Reptiles

17. (Fuxjager et al., 2014) - The geomagnetic environment in which sea turtle eggs incubate affects subsequent magnetic navigation behaviour of hatchlings

Two magnetic parameters used (inclination and intensity). Orientation assessed at a single virtual magnetic displacement. Possible locations with the given magnetic parameters do not appear near the suggested location (inclination value seems correct but total intensity around 3000 nT too high).

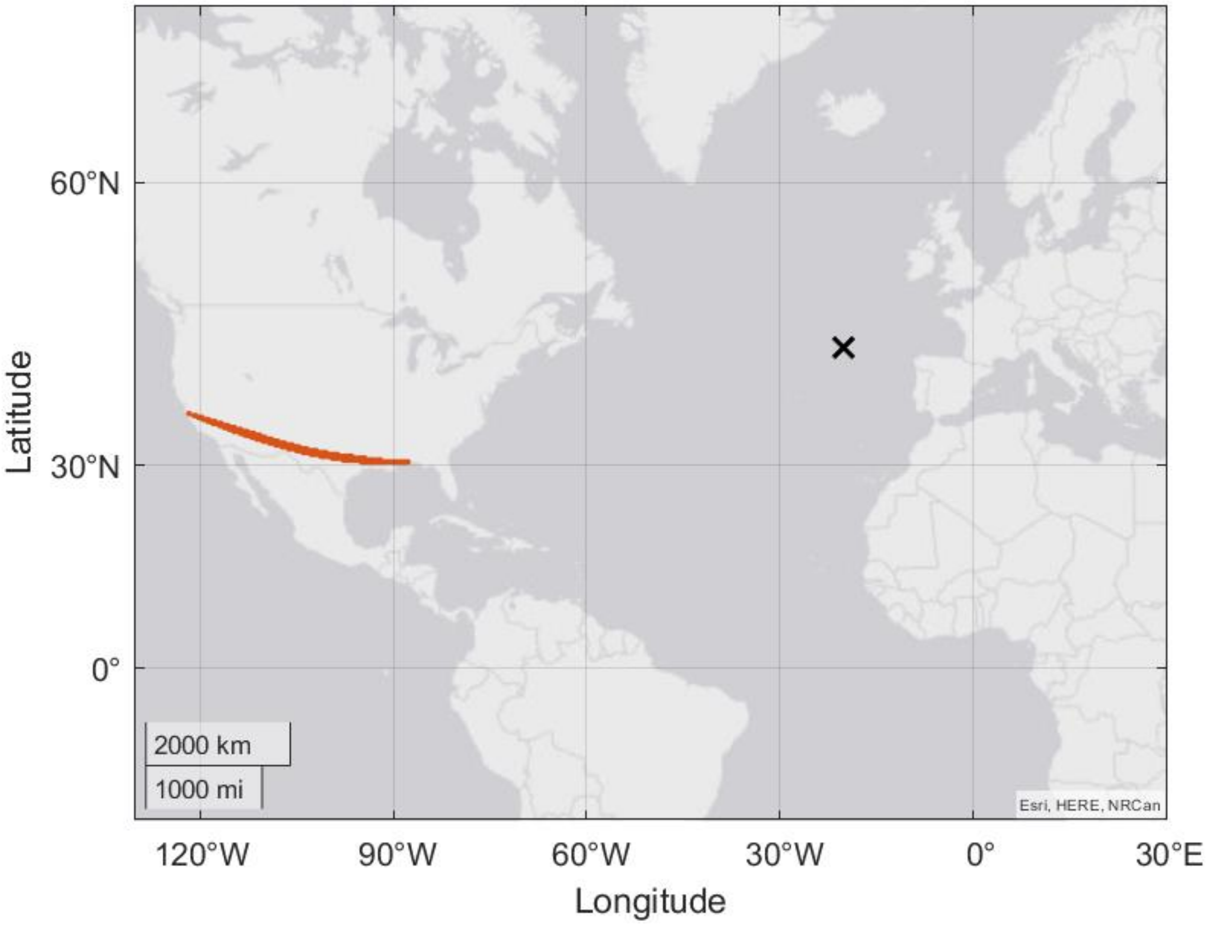

Supplementary Figure 15. Possible locations for the virtual magnetic displacement in Fuxjager et al., 2014. Virtual magnetic displacement location as suggested by the authors is indicated with 'x'. Possible locations with the same magnetic parameters as the virtual magnetic displacements and/or ambient conditions are highlighted in individual colours for each. The sensitivity used is ± 200 nT for total intensity, and ± 0.5° for inclination.

18. (Fuxjager et al., 2011) – in main article.
19. (Lohmann et al., 2004) – Geomagnetic map used in sea-turtle navigation

Two magnetic parameters used (inclination and intensity). Orientations assessed at two virtual magnetic displacements. Possible locations with the same magnetic parameters span areas East and West of both virtual displacements. Precise locations not given in paper.

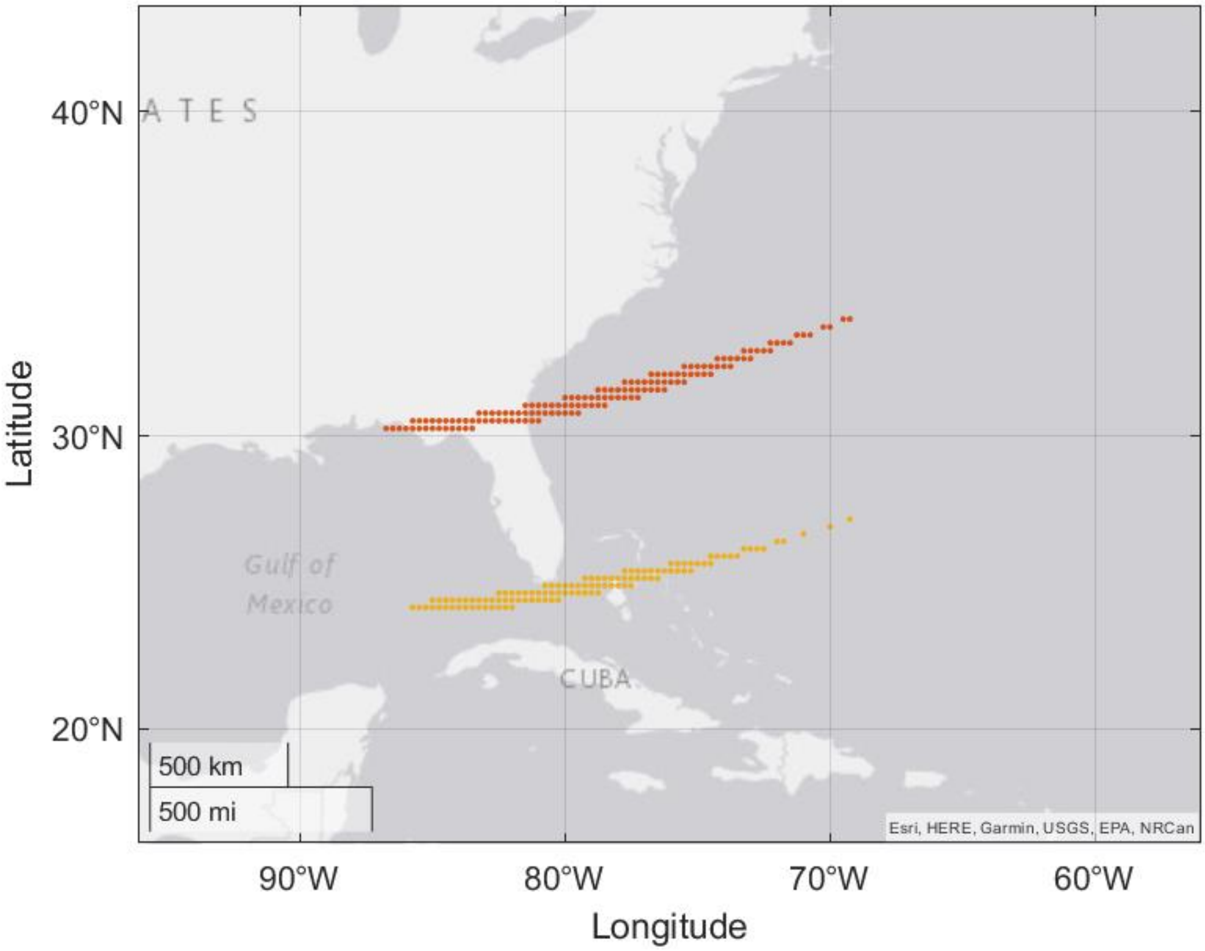

Supplementary Figure 16. Possible locations for the virtual magnetic displacements in Lohmann et al., 2004. Latitude and longitude values for the virtual magnetic displacement locations as are not detailed in the paper. Possible locations with the same magnetic parameters as the virtual magnetic displacements and/or ambient conditions are highlighted in individual colours for each. The sensitivity used is ± 200 nT for total intensity, and ± 0.5° for inclination.

20. (Lohmann et al., 2001) - Regional Magnetic Fields as Navigational Markers for Sea Turtles

Two magnetic parameters used (inclination and intensity). Orientations assessed at three virtual magnetic displacements. Possible locations for each span wide areas, and for the two Northern displacements they do not align exactly with the suggested location (rounding or updated IGRF model may be accountable). Possible locations for the South Atlantic displacement also appear in the South Pacific Ocean.

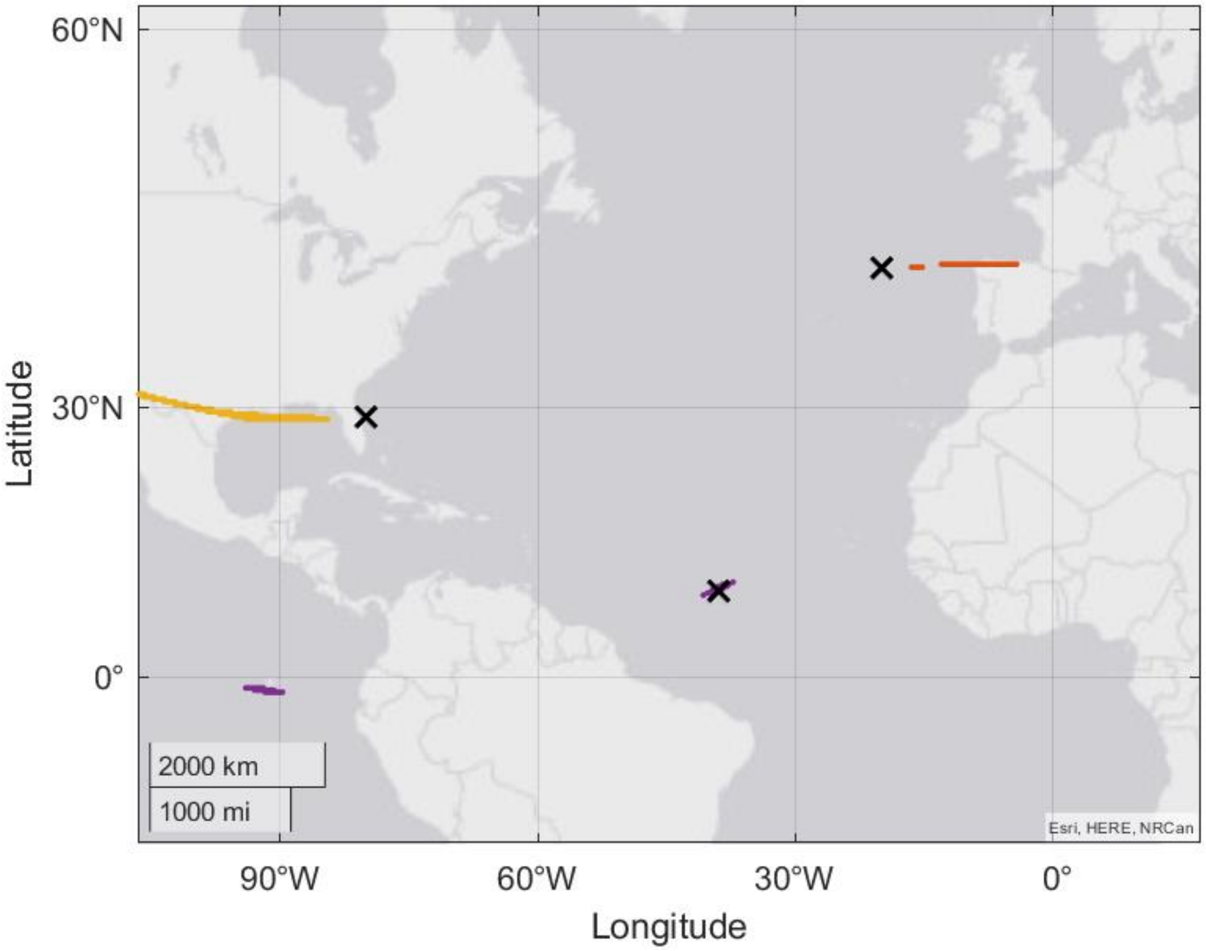

Supplementary Figure 17. Possible locations for the virtual magnetic displacements in Lohmann et al., 2001. Virtual magnetic displacement locations as suggested by the authors are indicated with ‘x’s. Possible locations with the same magnetic parameters as the virtual magnetic displacements and/or ambient conditions are highlighted in individual colours for each. The sensitivity used is ± 200 nT for total intensity, and ± 0.5° for inclination.

21. (Merrill and Salmon, 2011) - Magnetic orientation by hatchling loggerhead sea turtles (Caretta caretta) from the Gulf of Mexico

Two magnetic parameters used (inclination and intensity). Orientation assessed at seven virtual magnetic displacements. Exact suggested locations are not detailed in the text, but are indicated on a figure in the manuscript. Possible locations with the same magnetic parameters as the virtual displacements span wide areas, sometimes overlap, and are not all near to the suggested locations.

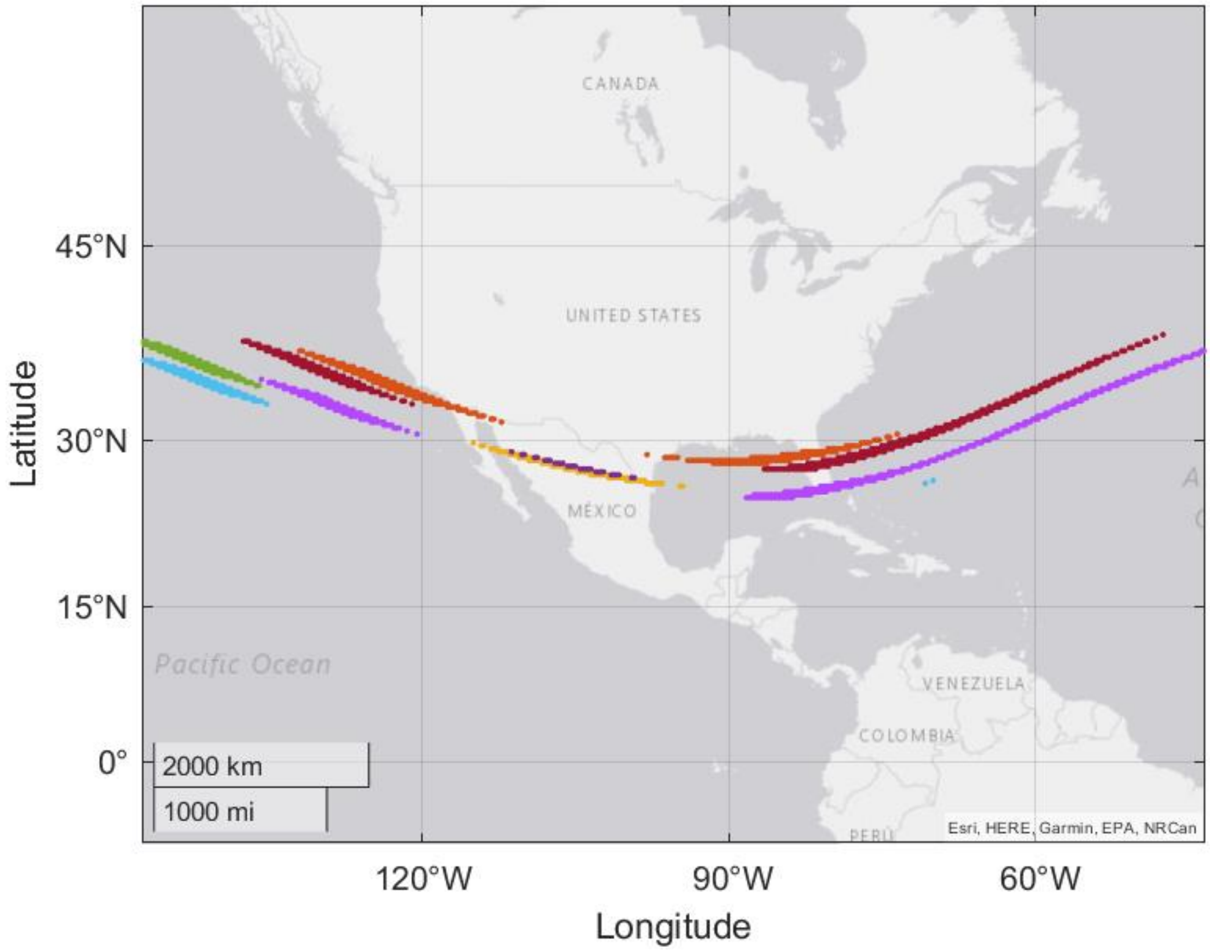

Supplementary Figure 18. Possible locations for the virtual magnetic displacements in Merrill and Salmon et al., 2011. Latitude and longitude values for the virtual magnetic displacement locations were not detailed in the paper. Possible locations with the same magnetic parameters as the virtual magnetic displacements and/or ambient conditions are highlighted in individual colours for each. The sensitivity used is ± 200 nT for total intensity, and ± 0.5° for inclination.

22. (Putman et al., 2015) - Magnetic navigation behavior and the oceanic ecology of young loggerhead sea turtles.

Two magnetic parameters used (inclination and intensity). Orientations assessed at five virtual magnetic displacements. Possible locations span wide areas including, for the Southern Spain displacement, possible locations also in the Gulf of Mexico.

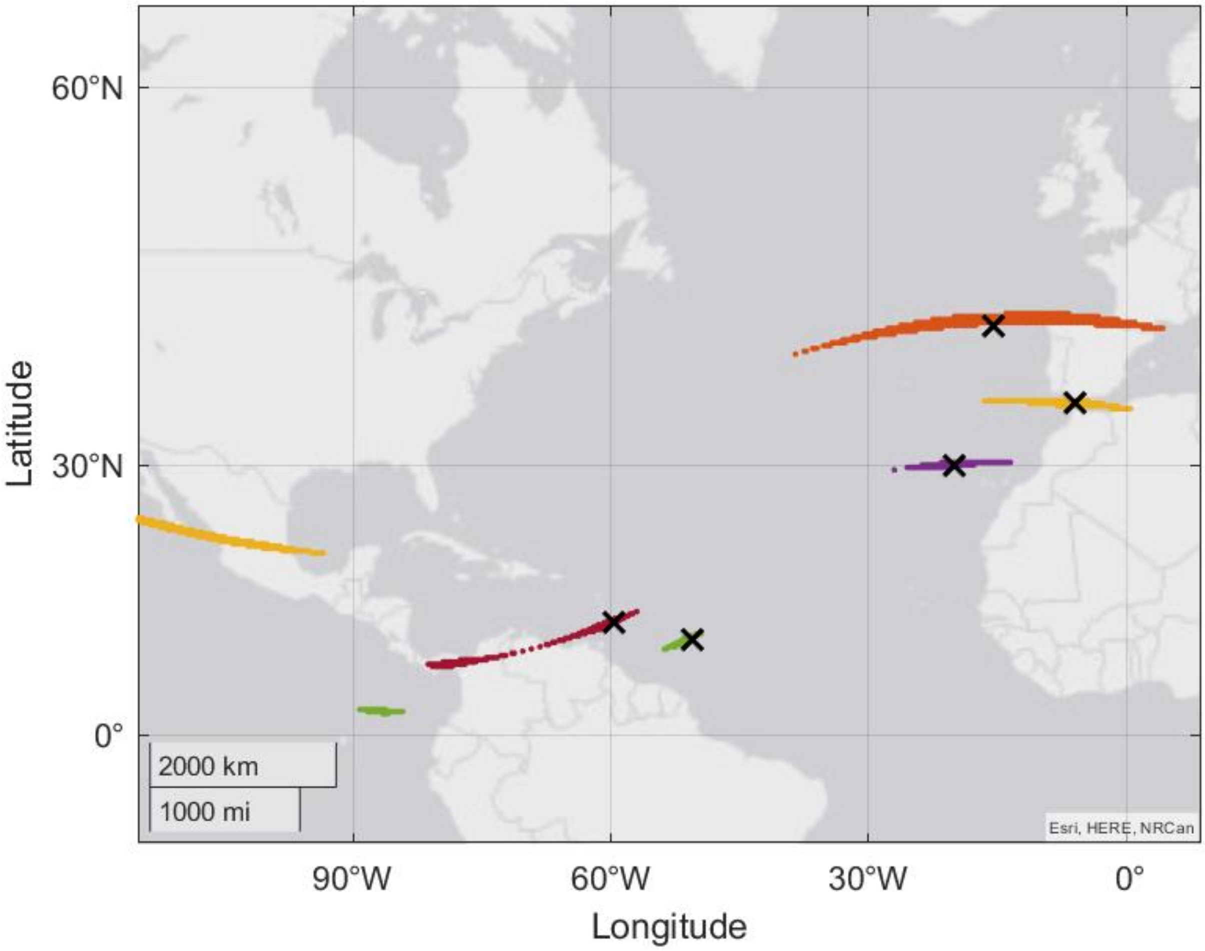

Supplementary Figure 19. Possible locations for the virtual magnetic displacements in Putman et al., 2015. Virtual magnetic displacement locations as suggested by the authors are indicated with 'x's. Possible locations with the same magnetic parameters as the virtual magnetic displacements and/or ambient conditions are highlighted in individual colours for each. The sensitivity used is ± 200 nT for total intensity, and ± 0.5° for inclination.

23. (Putman et al., 2011) – in main article.
24. (Boles and Lohmann, 2003) - True navigation and magnetic maps in spiny lobsters

Two magnetic parameters used (inclination and intensity). Orientation assessed at two virtual magnetic displacements. Possible locations span areas in the Gulf of Mexico and into the Atlantic Ocean for the Northern displacement, and in the Caribbean Sea and a large stretch of the Mid-Atlantic Ocean for the Southern displacement.

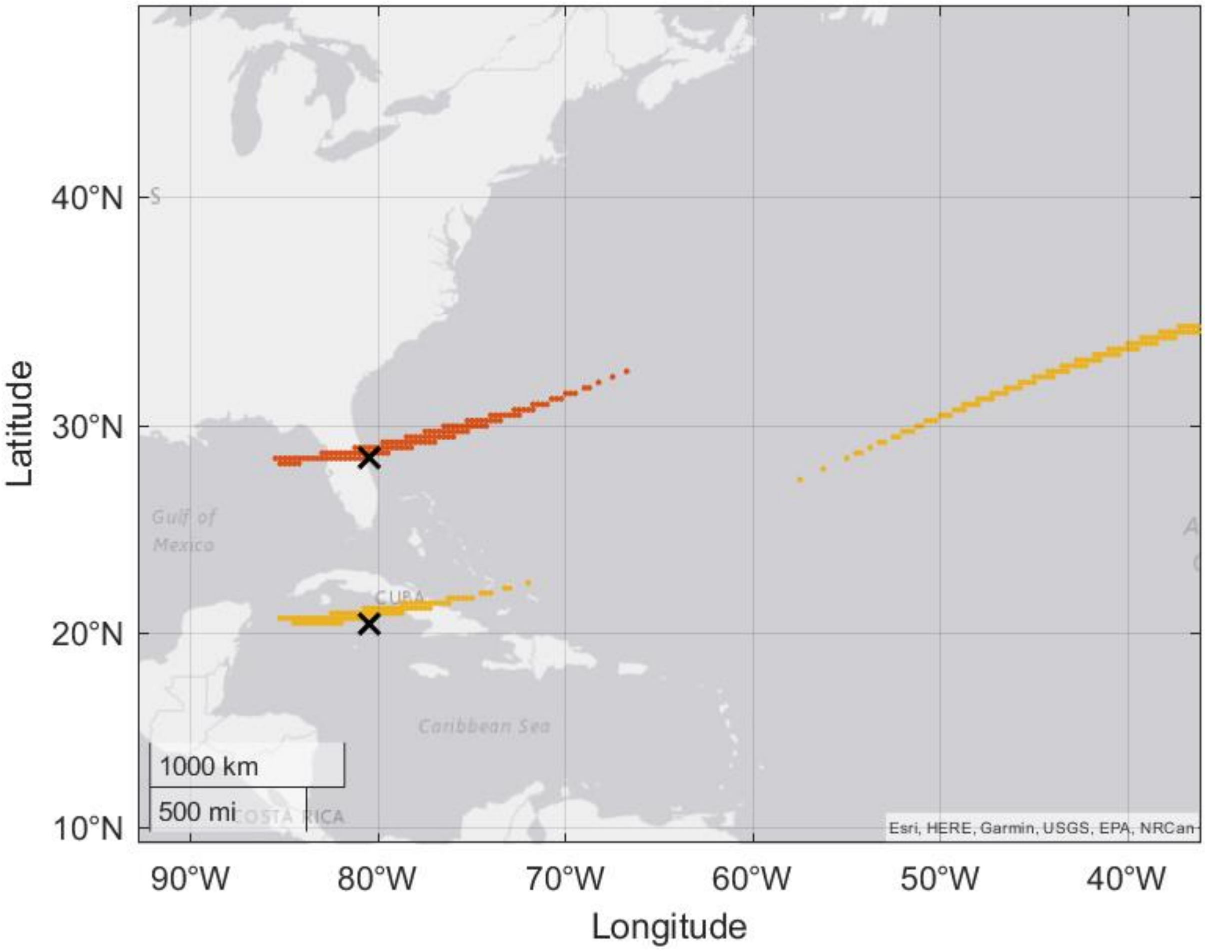

Supplementary Figure 20. Possible locations for the virtual magnetic displacements in Boles and Lohmann et al., 2003. Virtual magnetic displacement locations as suggested by the authors are indicated with 'x's. Possible locations with the same magnetic parameters as the virtual magnetic displacements and/or ambient conditions are highlighted in individual colours for each. The sensitivity used is ± 200 nT for total intensity, and ± 0.5° for inclination.

Supplementary References

Boles LC, Lohmann KJ. 2003. True navigation and magnetic maps in spiny lobsters **421**:60–63. doi:10.1038/nature01226

Boström JE, Fransson T, Henshaw I, Jakobsson S, Kullberg C, Åkesson S. 2010. Autumn migratory fuelling: A response to simulated magnetic displacements in juvenile wheatears, Oenanthe oenanthe. *Behav Ecol Sociobiol* **64**:1725–1732. doi:10.1007/s00265-010-0985-1

Boström JE, Kullberg C, Åkesson S. 2012. Northern magnetic displacements trigger endogenous fuelling responses in a naive bird migrant. *Behav Ecol Sociobiol* **66**:819–821. doi:10.1007/s00265-012-1333-4

Bulte M, Heyers D, Mouritsen H, Bairlein F. 2017. Geomagnetic information modulates nocturnal migratory restlessness but not fueling in a long distance migratory songbird. *J Avian Biol* **48**:75–82. doi:https://doi.org/10.1111/jav.01285

Fuxjager MJ, Davidoff KR, Mangiamele LA, Lohmann KJ. 2014. The geomagnetic environment in which sea turtle eggs incubate affects subsequent magnetic navigation behaviour of hatchlings. *Proc R Soc London Ser B Biol Sci*. doi:10.1098/rspb.2014.1218

Fuxjager MJ, Eastwood BS, Lohmann KJ. 2011. Orientation of hatchling loggerhead sea turtles to regional magnetic fields along a transoceanic migratory pathway. *J Exp Biol* **214**:2504–2508. doi:10.1242/jeb.055921

Henshaw I, Fransson T, Jakobsson S, Jenni-Eiermann S, Kullberg C. 2009. Information from the geomagnetic field triggers a reduced adrenocortical response in a migratory bird. *J Exp Biol* **212**:2902–2907. doi:10.1242/JEB.033332

Henshaw I, Fransson T, Jakobsson S, Kullberg C. 2010. Geomagnetic field affects spring migratory direction in a long distance migrant. *Behav Ecol Sociobiol* **64**:1317–1323. doi:10.1007/S00265-010-0946-8

Henshaw I, Fransson T, Jakobsson S, Lind J, Vallin A, Kullberg C. 2008. Food intake and fuel deposition in a migratory bird is affected by multiple as well as single-step changes in the magnetic field. *J Exp Biol* **211**:649–653. doi:10.1242/JEB.014183

Ilieva M, Bianco G, Åkesson S. 2018. Effect of geomagnetic field on migratory activity in a diurnal passerine migrant, the dunnock, Prunella modularis. *Anim Behav* **146**:79–85. doi:10.1016/J.ANBEHAV.2018.10.007

Ilieva M, Bianco G, Åkesson S. 2016. Does migratory distance affect fuelling in a medium-distance passerine migrant?: results from direct and step-wise simulated magnetic displacements. *Biol Open* **5**:272–278. doi:10.1242/bio.014779

Keller BA, Putman NF, Grubbs RD, Portnoy DS, Murphy TP. 2021. Map-like use of Earth's magnetic field in sharks. *Curr Biol* 2881-2886.e3.

Kullberg C, Henshaw I, Jakobsson S, Johansson P, Fransson T. 2007. Fuelling decisions in migratory birds: geomagnetic cues override the seasonal effect. *Proc R Soc B Biol Sci* **274**:2145–2151. doi:10.1098/RSPB.2007.0554

Kullberg C, Lind J, Fransson T, Jakobsson S, Vallin A. 2003. Magnetic cues and time of season affect fuel deposition in migratory thrush nightingales (Luscinia luscinia). *Proc R Soc London Ser B Biol Sci* **270**:373–378. doi:10.1098/RSPB.2002.2273

Lohmann KJ, Cain SD, Dodge SA, Lohmann CMF. 2001. Regional Magnetic Fields as Navigational

Markers for Sea Turtles. *Science (80-)* **294**:364–366. doi:10.1126/SCIENCE.1064557

Lohmann KJ, Lohmann CMF, Ehrhart LM, Bagley DA, Swing T. 2004. Geomagnetic map used in sea-turtle navigation. *Nat 2004 4286986* **428**:909–910. doi:10.1038/428909a

Merrill MW, Salmon M. 2011. Magnetic orientation by hatchling loggerhead sea turtles (Caretta caretta) from the Gulf of Mexico. *Mar Biol* **158**:101–112. doi:10.1007/S00227-010-1545-Y/FIGURES/5

Naisbett-Jones LC, Putman NF, Stephenson JF, Ladak S, Young KA. 2017. A Magnetic Map Leads Juvenile European Eels to the Gulf Stream. *Curr Biol* **27**:1236–1240. doi:10.1016/j.cub.2017.03.015

Putman NF, Endres CS, Lohmann CMF, Lohmann KJ. 2011. Longitude perception and bicoordinate magnetic maps in sea turtles. *Curr Biol* **21**:463–466. doi:10.1016/j.cub.2011.01.057

Putman NF, Meinke AM, Noakes DLG. 2014a. Rearing in a distorted magnetic field disrupts the 'map sense' of juvenile steelhead trout. *Biol Lett* **10**. doi:10.1098/RSBL.2014.0169

Putman NF, Scanlan MM, Billman EJ, O'Neil JP, Couture RB, Quinn TP, Lohmann KJ, Noakes DLG. 2014b. An Inherited Magnetic Map Guides Ocean Navigation in Juvenile Pacific Salmon. *Curr Biol* **24**:446–450. doi:10.1016/J.CUB.2014.01.017

Putman NF, Verley P, Endres CS, Lohmann KJ. 2015. Magnetic navigation behavior and the oceanic ecology of young loggerhead sea turtles. *J Exp Biol* **218**:1044–1050. doi:10.1242/jeb.109975

Putman NF, Williams CR, Gallagher EP, Dittman AH. 2020. A sense of place: Pink salmon use a magnetic map for orientation. *J Exp Biol* **223**. doi:10.1242/jeb.218735

Scanlan MM, Putman NF, Pollock AM, Noakes DLG. 2018. Magnetic map in nonanadromous Atlantic salmon **23**:10995–10999. doi:10.1073/pnas.1807705115
