# Supplementary material for: Sense of doubt: inaccurate and alternate locations of virtual magnetic displacements may give a distorted view of animal magnetoreception ability
Source: Commun Biol. 2023 Feb 20;6:187. doi: 10.1038/s42003-023-04530-w (PMC9941108; doi:10.1038/s42003-023-04530-w)
Supplement: Supplementary file 3 — Description of Additional Supplementary Data [file 42003_2023_4530_MOESM3_ESM.docx]

**Description of Additional Supplementary Files**

**File name:** Supplementary Data 1

**Description:** The source magnetic parameter values behind the graphs in the paper
